# Risk Assessment for Longitudinal Trajectories of Modifiable Lifestyle Factors on Chronic Kidney Disease Burden in China: A Population-based Study

**DOI:** 10.2188/jea.JE20200497

**Published:** 2022-10-05

**Authors:** Ping Li, Mingjia Yang, Dong Hang, Yongyue Wei, Hongling Di, Hongbing Shen, Zhihong Liu

**Affiliations:** 1 National Clinical Research Center of Kidney Diseases, Jinling Clinical Medical College of Nanjing Medical University, Nanjing, China; 2 Department of Epidemiology and Biostatistics, Jiangsu Key Lab of Cancer Biomarkers, Prevention and Treatment, Collaborative Innovation Center for Cancer Personalized Medicine, School of Public Health, Nanjing Medical University, Nanjing, China; 3 National Clinical Research Center of Kidney Diseases, Jinling Hospital, Nanjing University School of Medicine, Nanjing, China

**Keywords:** chronic kidney disease, lifestyle, longitudinal trajectories, comparative risk assessment

## Abstract

**Background:**

Chronic kidney disease (CKD) is an important contributor to morbidity and mortality from noncommunicable diseases. We aimed to examine the longitudinal trajectories in risk factors, estimate their impact on CKD burden in China from 1991 to 2011, and project trends in the next 20 years.

**Methods:**

We used data from a cohort of the China Health and Nutrition Survey and applied the comparative risk assessment method to estimate the number of CKD events attributable to all non-optimal levels of each risk factors.

**Results:**

In 2011, current smoking was the leading individual attributable factor for CKD burden in China responsible for 7.9 (95% confidence interval [CI], 7.5–8.3) million CKD cases with a population-attributable fraction of 8.7% (95% CI, 6.0–11.6), while the rates of smoking have reduced and may have mitigated the increase in CKD. High triglyceride (TG) and high systolic blood pressure (SBP) were the leading metabolic risk factors responsible for 6.8 (95% CI, 6.4–7.1) million and 5.8 (95% CI, 5.5–6.1) million CKD-attributable cases, respectively. Additionally, the number of CKD cases associated with high body mass index (BMI), high diastolic blood pressure (DBP), high plasma glucose, and low high-density lipoprotein cholesterol (HDL-C) was 5.4 (95% CI, 5.1–5.6), 3.9 (95% CI, 3.7–4.1), 3.0 (95% CI, 2.8–3.1) and 2.6 (95% CI, 2.5–2.8) million, respectively.

**Conclusion:**

Current smoking, high TG, and high SBP were the top three risk factors that contributed to CKD burden in China. Increased BMI, DBP, plasma glucose, and decreased HDL-C were also associated with the increase in CKD burden.

## INTRODUCTION

With the rapid development of social economy and the improvement of living standard, people’s lifestyles are changing. Increasing evidence suggests that lifestyles play important role in the prevention and development of non-communicable diseases, especially diabetes, obesity, metabolic syndrome, cardiovascular diseases, and tumor, all of which are the risk factors for the occurrence and development of chronic kidney disease (CKD). CKD is an important contributor to cardiovascular events, morbidity, and mortality, and this disease has been a worldwide public health problem. The global prevalence of CKD is about 8–16% and still increasing dramatically.^1^ In 2017, the global cases of all-stage CKD were 697.5 million and the all-age prevalence of CKD increased 29.3% since 1990. Furthermore, the global all-age mortality rate from CKD increased 41.5% between 1990 and 2017.^2^ The Global Burden of Disease, Injuries, and Risk Factors Study (GBD) ranks CKD as the 12th leading cause of death out of 133 conditions.^3^

China is the world’s largest developing country. In the past 4 decades, China has seen rapid demographic and epidemiological transitions, along with accelerated urbanization and industrialization, led to a dramatic shift in diet from traditional to western dietary patterns and a steep decline in physical activity levels. Fundamental transformations in overall population health have occurred and are continuing. In 2017, high systolic blood pressure (SBP), smoking, and high-sodium diet were among the leading three risk factors contributing to deaths and disability-adjusted life-years (DALYs) in China.^4^ Yanping Li et al found that high blood pressure, increased body mass index (BMI), decreased physical activity, smoking, and unhealthy dietary factors contribute to the burden of cardiovascular disease and diabetes in China.^5^^,^^6^ Previous studies have shown that CKD is largely preventable and treatable: adherence to the healthy dietary patterns, physical activity, and not smoking were associated with a lower risk of incident CKD.^7^^–^^10^ However, there has been no comprehensive estimation of the longitudinal trajectories of lifestyle factors and their related CKD burden up to now.

Therefore, we use the data from an ongoing open cohort of the China Health and Nutrition Survey (CHNS)^11^ to describe the time trends in lifestyle risk factors related to CKD from 1991 to 2011, and estimate the number of CKD cases attributable to suboptimal levels of these risk factors, to evaluate current public health policies, provide guidance for future CKD prevention and health promotion, and promote the Healthy China 2030 plan.

## METHODS

### Study design and population

Data were derived from CHNS, which was a nationally longitudinal study covered nine provinces in China since 1989 (representing 553 million people). Details about CHNS study have been reported elsewhere.^6^ Briefly, the survey uses a multistage random-cluster sampling process to select samples in both urban and rural areas. All the members in the selected household were invited to participate in the study. The survey was approved by the institutional review committees of the University of North Carolina and the National Institute of Nutrition and Food Safety.

In present study, we included eight rounds of data collection (1991, 1993, 1997, 2000, 2004, 2006, 2009, and 2011) and excluded participants who were <18 years of age or pregnant at the time of the survey. Data are available at http://www.cpc.unc.edu/projects/china.

Information on demographic characteristics and lifestyle factors was obtained by well-trained staff using a structured questionnaire in each wave. Height, weight, and blood pressure were measured using standard procedures, and BMI was calculated as weight (kg) divided by height squared (m^2^); blood pressure was measured three times, and the mean value of three measurements was used. Laboratory indicators, including blood glucose, triglyceride (TG), and high-density lipoprotein cholesterol (HDL-C), were measured using standard laboratory procedures with methods of the glucose oxidase phenol 4-aminoantipyrine peroxidase, glycerol phosphate oxidase-p-aminophenazone, and enzymatic, respectively.

### Risk factors selection

In this comparative risk assessment, we selected risk factors of CKD based on the following criteria: 1) sufficient evidence suggested an association with CKD; 2) could be intervened; 3) exposure data were available in study population. A total of seven factors were included: high SBP, high diastolic blood pressure (DBP), high BMI, current smoking, high TG, high glucose, and low HDL-C. Because only one measurement of blood indicators was available, we included the seven risk factors for CKD burden estimate in 2011 and four non-blood factors for time trend analysis.

### CKD cases

We extracted the prevalence with corresponding 95% confidence interval (CI) of Chinese CKD in 2010 from a published paper covering 47,204 participants.^12^ Total CKD cases were obtained by multiply the prevalence of CKD by the population number according to 2010 population census in China.

### Attributable burden estimate

Relative risks (RRs) of risk factors for CKD were extracted from the most recent high-quality reviews or meta-analyses in China. When unavailable, we expanded the scope to Asia or other regions.

To assess the proportion of CKD cases attributable to non-optimal levels of exposures, we used theoretical minimum risk exposure distribution (TMRED). In the comparative risk assessment framework, disease burden attributable to risk factors is calculated with reference to an alternative (counterfactual) distribution of exposure, termed the TMRED.^13^^,^^14^ In present study, the TMRED for current smoking was no smoking. For other six risk factors, whose exposure of zero was impossible, the TMRED was set by levels with the lowest risk in epidemiological studies. The TMRED and sources of RRs of risk factors included are shown in Table 1. For categorical exposure, we calculated attributable burden according to the following formula, where population-attributable fraction (PAF) Pi is the fraction of the population in exposure category i, RRi is the RR for exposure category i, and n is the number of exposure categories.

$$PAF=\frac{\sum_{i=1}^{n}P_{i}({RR}_{i}-1)}{\sum_{i=1}^{n}P_{i}({RR}_{i}-1)+1}$$

**Table 1.** Sources and magnitudes of RRs for the effects of CKD

Risk factors	TMRED (SD)	RR of CKD		

		Sources	Exposure metric, units	RR (95% CI)
High BMI	21 (1) kg/m^2^	Garofalo C et al. Kidney Int. (2017)	BMI per kg/m^2^ increase	1.02 (1.01–1.03)
High blood pressure (SBP)	115 (6) mm Hg	Garofalo C et al. Am J Kidney Dis. (2016)	SBP per 10 mm Hg increase	1.08 (1.04–1.11)
High blood pressure (DBP)	75 (6) mm Hg	Garofalo C et al. Am J Kidney Dis. (2016)	DBP per 10 mm Hg increase	1.12 (1.04–1.20)
Smoking	Never	Xia J et al. Nephrol Dial Transplant. (2017)	Ever smoking vs Never smoking	1.27 (1.19–1.35)
			Current smoking vs Never smoking	1.34 (1.23–1.47)
High TG	TG <1.7 mmol/L	Rashidbeygi E et al. Diabetes & Metabolic Syndrome (2019)	TG >1.7 mmol/L	1.32 (1.15–1.51)
low HDL-C	Female >0.9 mmol/L	Rashidbeygi E et al. Diabetes & Metabolic Syndrome (2019)	Female <0.9 mmol/L	1.24 (1.04–1.47)
	Male >1.1 mmol/L		Male <1.1 mmol/L	
High plasma glucose	Plasma glucose <5.6 mmol/L	Rashidbeygi E et al. Diabetes & Metabolic Syndrome (2019)	Plasma glucose >5.6 mmol/L	1.89 (1.51–2.37)

BMI, body mass index; CKD, chronic kidney disease; DBP, diastolic blood pressure; HDL-C, high-density lipoprotein cholesterol; RR, relative risk; SBP, systolic blood pressure; SD, standard deviation; TG, triglyceride; TMRED, theoretical minimum risk exposure distribution.

For continuous exposure, we calculated attributable burden according to the following formula, where RR(x) is the RR at exposure level x, P1 (x) is the population distribution of exposure, P2 (x) is the counterfactual distribution of the theoretical minimum risk exposure, and m is the maximum exposure level.

$$PAF=\frac{\int_{x=0}^{m}RR(x)P1(x)dx-\int_{x=0}^{m}RR(x)P2(x)dx}{\int_{x=0}^{m}RR(x)P1(x)dx}$$

### Statistical analysis

Mean and standard error (SE) or percentage of each risk factor was calculated by age, gender, and residence in each wave, and general linear mixed model was used to calculate covariate-adjusted means with adjustment for age, gender, residence, education, occupation, and provinces. To assess time trends of each risk factor, the year of each wave was included in the model as a scored variable.

We standardized overall distribution of each risk factor in the joint classifications of age, sex, and residence in each wave using data from the 2010 Chinese Population Census as the reference. The number of CKD cases attributable to each risk factor was calculated by multiplying its PAF by the total CKD cases. Future trends prediction of four risk factors (SBP, DBP, BMI, current smoking) for 2013–2031 was conducted using a random-effects model within each stratum of age, sex, and residence among participants who completed at least three surveys during 1991–2011. We performed the analysis using SAS 9.4 (SAS Institute, Cary, NC, USA).

## RESULTS

### Longitudinal trajectories of risk factors

Table 2 presents the mean SBP increased over time in both genders, residence (rural/urban), and different age groups except for those aged 70 or older. Similarly, the mean DBP increased over time in all subgroups except for those aged 60 or older. In contrast, the proportion of current smoking decreased over time in all subgroups except for those aged 60 or older. The mean BMI increased over time in all strata of age, sex, and residence.

**Table 2.** Distribution of the risk factors for CKD over time^a^

	Sex		Age, years				Residence	
		
	Male	Female	18–39	40–59	60–69	≥70	Urban	Rural
Systolic Blood Pressure, mm Hg								
1991	116 (1.1)	112 (1.2)	109 (1.2)	117 (1.2)	127 (1.3)	133 (1.5)	115 (1.2)	113 (1.2)
1993	121 (1.2)	117 (1.2)	115 (1.2)	121 (1.2)	133 (1.3)	138 (1.5)	120 (1.2)	119 (1.2)
1997	119 (2.3)	114 (2.3)	113 (2.3)	119 (2.3)	129 (2.4)	133 (2.6)	116 (2.3)	117 (2.3)
2000	122 (1.2)	117 (1.2)	115 (1.2)	122 (1.2)	131 (1.4)	136 (1.8)	119 (1.2)	120 (1.2)
2004	120 (2.3)	115 (2.3)	113 (2.3)	119 (2.3)	127 (2.4)	131 (2.8)	118 (2.3)	118 (2.3)
2006	119 (1.6)	114 (1.6)	112 (1.6)	118 (1.6)	125 (1.8)	128 (2.2)	117 (1.6)	117 (1.6)
2009	121 (1.5)	117 (1.6)	112 (1.6)	120 (1.5)	128 (1.7)	130 (2.1)	118 (1.6)	120 (1.5)
2011	124 (0.7)	119 (0.7)	116 (0.7)	123 (0.7)	129 (1.0)	129 (1.5)	121 (0.7)	122 (0.7)
*P* for trend	<0.001	<0.001	<0.001	<0.001	<0.001	0.708	<0.001	<0.001
Diastolic Blood Pressure, mm Hg								
1991	76 (0.8)	73 (0.8)	72 (0.8)	76 (0.8)	79 (0.9)	81 (1.0)	74 (0.8)	74 (0.8)
1993	79 (0.8)	76 (0.8)	75 (0.8)	79 (0.8)	82 (0.9)	83 (1.1)	78 (0.8)	77 (0.8)
1997	78 (1.5)	76 (1.5)	75 (1.5)	79 (1.6)	82 (1.6)	82 (1.8)	77 (1.5)	77 (1.5)
2000	80 (0.8)	77 (0.8)	76 (0.8)	80 (0.8)	81 (0.9)	82 (1.2)	78 (0.8)	78 (0.8)
2004	79 (1.6)	75 (1.6)	75 (1.6)	79 (1.6)	80 (1.7)	77 (2.0)	77 (1.6)	77 (1.6)
2006	80 (1.1)	76 (1.1)	76 (1.1)	79 (1.1)	80 (1.2)	80 (1.5)	78 (1.1)	78 (1.1)
2009	81 (1.3)	78 (1.3)	76 (1.3)	81 (1.3)	81 (1.4)	81 (1.6)	79 (1.3)	79 (1.3)
2011	80 (0.5)	77 (0.5)	76 (0.5)	80 (0.5)	80 (0.6)	77 (1.0)	78 (0.5)	79 (0.5)
*P* for trend	<0.001	<0.001	<0.001	<0.001	0.044	0.605	<0.001	<0.001
Body Mass Index, kg/m^2^								
1991	21.8 (0.2)	22.3 (0.2)	21.8 (0.2)	22.5 (0.2)	22.1 (0.2)	21.4 (0.2)	22.2 (0.2)	21.9 (0.2)
1993	22.0 (0.2)	22.5 (0.2)	21.9 (0.2)	22.8 (0.2)	22.5 (0.2)	21.8 (0.3)	22.4 (0.2)	22.1 (0.2)
1997	22.9 (0.4)	23.3 (0.4)	22.8 (0.4)	23.5 (0.4)	22.6 (0.5)	22.5 (0.5)	23.2 (0.4)	23.0 (0.4)
2000	23.5 (0.2)	23.8 (0.2)	23.2 (0.2)	24.1 (0.2)	23.1 (0.3)	22.8 (0.3)	23.8 (0.2)	23.5 (0.2)
2004	22.8 (0.5)	22.8 (0.5)	22.4 (0.4)	23.3 (0.5)	22.5 (0.5)	21.6 (0.6)	22.9 (0.5)	22.8 (0.5)
2006	23.5 (0.3)	23.5 (0.3)	23.1 (0.3)	23.9 (0.3)	22.9 (0.4)	22.6 (0.4)	23.4 (0.3)	23.6 (0.3)
2009	23.2 (0.3)	23.1 (0.3)	22.5 (0.3)	23.6 (0.3)	22.7 (0.3)	21.7 (0.4)	23.2 (0.3)	23.2 (0.3)
2011	24.0 (0.2)	23.6 (0.2)	23.1 (0.2)	24.2 (0.2)	23.3 (0.3)	23.8 (0.4)	23.7 (0.2)	23.8 (0.2)
*P* for trend	<0.001	<0.001	<0.001	<0.001	<0.001	<0.001	<0.001	<0.001
Current Smoking, %^b^								
1991	66.7	4.3	31.9	37.6	35.5	25.5	32.7	34.2
1993	64.4	4.4	30.6	36.7	34.5	25.5	32.2	33.1
1997	58.4	4.2	29.7	35.7	29	20.5	29.9	31.8
2000	58.6	4.5	28.9	33.6	28.4	23.1	28.9	31
2004	56.0	4.0	27.8	31.9	28.2	21.7	28.1	29.6
2006	53.5	3.5	26	30.2	27.5	18.5	26.9	27.5
2009	54.6	3.6	27.7	30.0	28.2	20.9	26.6	29
2011	52.7	2.9	24.3	29.5	24.7	21.7	24.7	27.7
*P* for trend	<0.001	<0.001	<0.001	<0.001	0.026	0.192	<0.001	<0.001

CKD, chronic kidney disease.

^a^We applied the multivariate-adjusted general linear mixed regression model to calculate covariate-adjusted mean levels with adjustment for age, gender, residence, education, occupation, and provinces; to quantify time trends of the risk factors, the regression models included the year of each wave as a scored trend variable.

^b^Values are mean (SE) except current smoking, which is prevalence (%).

### Attributable burden of CKD

**Figure 1.**
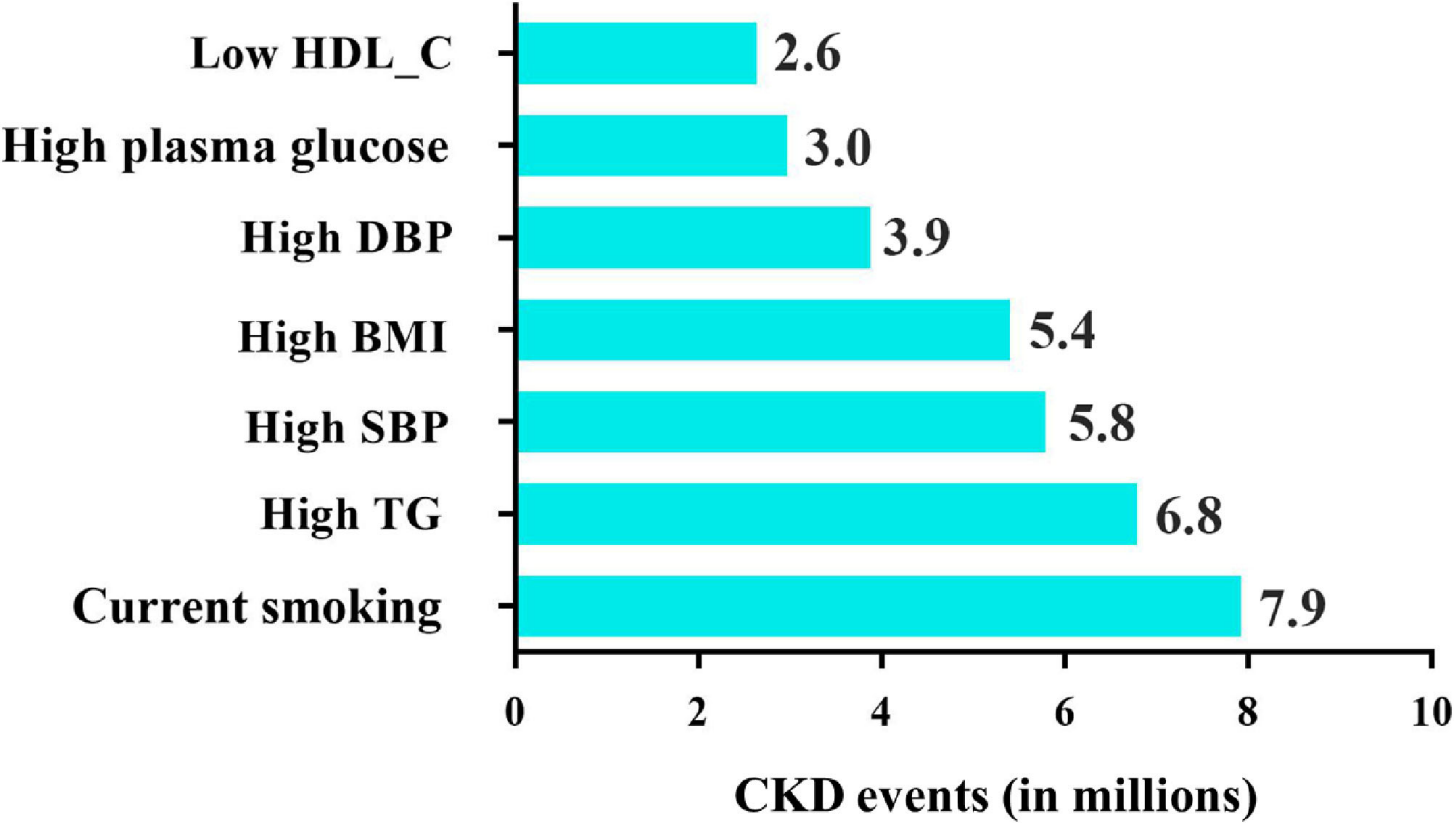
CKD cases attributable to 7 risk factors in 2011. CKD events represented as solid bars. Data of plasma glucose, HDL-C and TG were collected in 2009 and carried forward for estimation of CKD burden in 2011. BMI, body mass index; CKD, chronic kidney disease; DBP, diastolic blood pressure; HDL-C, high-density lipoprotein cholesterol; SBP, systolic blood pressure; TG, triglyceride.

According to the population census of China in 2010, there were 846,662,309 individuals older than 18, the distribution of Chinese population according gender, residence, and age have been displayed in eTable 1. The average prevalence of CKD in China was 10.8% (95% CI, 10.2–11.3%). The estimated number of CKD events among Chinese adults in 2011 was 91.4 (95% CI, 86.4–95.7) million. Figure 1 presents current smoking, high TG, and high SBP were the top three risk factors that contributed to CKD burden in China. Current smoking had an estimated PAF of 8.7% (95% CI, 6.0–11.6%) and accounted for 7.9 (95% CI, 7.5–8.3) million CKD cases in 2011; high TG with a PAF of 7.4% (95% CI, 3.2–12.0%) was responsible for 6.8 (95% CI, 6.4–7.1) million cases; and high SBP had a PAF of 6.3% (95% CI, 3.2–8.7%) and contributed to 5.8 (95% CI, 5.5–6.1) million cases.

For the other risk factors, high BMI with a PAF of 5.9% (95% CI, 2.9–8.8%) was responsible for 5.4 (95% CI, 5.1–5.6) million cases; high DBP showed a PAF of 4.2% (95% CI, 1.4–7.0%) and accounted for 3.9 (95% CI, 3.7–4.1) million cases; in addition, low HDL-C with a PAF of 2.9% (95% CI, 1.5–4.4%) and high glucose with a PAF of 3.3% (95% CI, 0.7–5.9%) contributed to 2.6 (95% CI, 2.5–2.8) million and 3.0 (95% CI, 2.8–3.1) million cases, respectively.

### Future trends prediction of CKD burden

**Figure 2.**
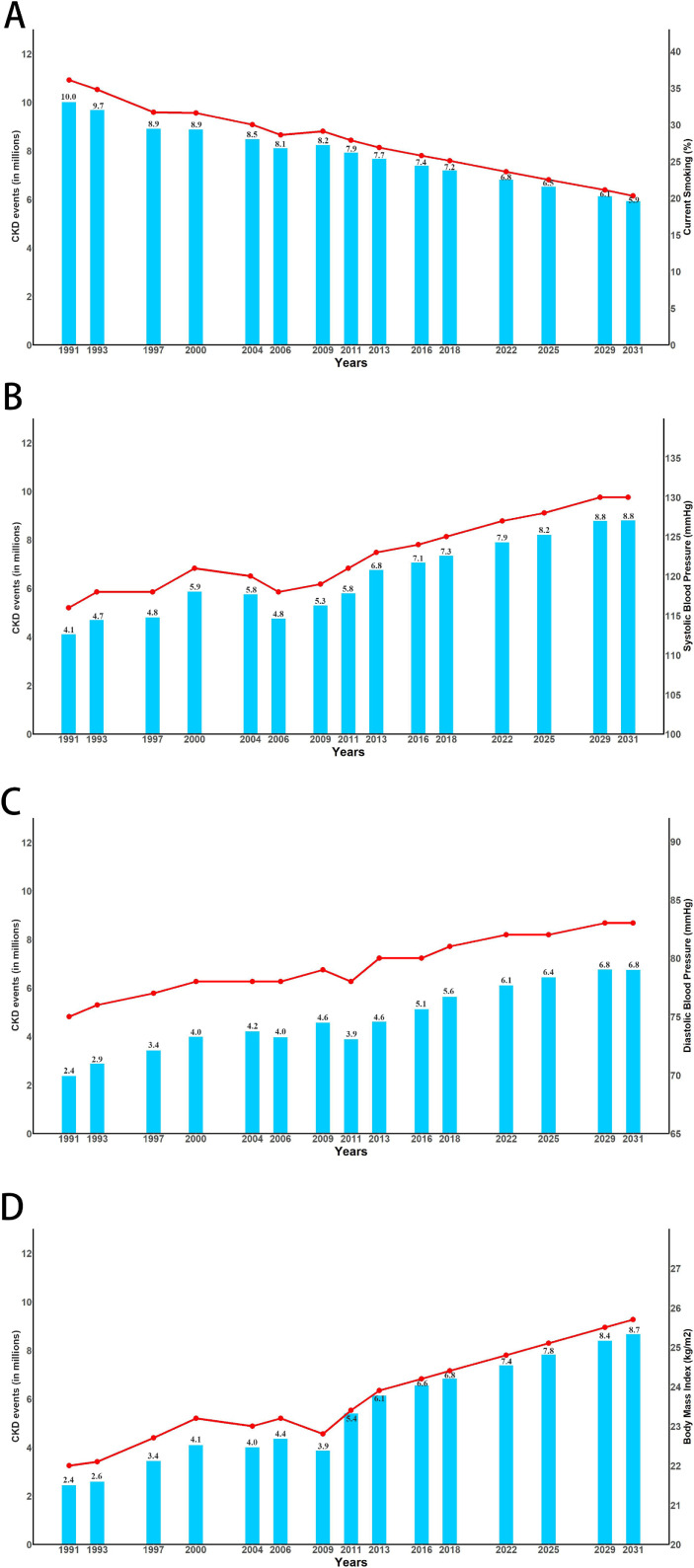
Longitudinal trajectories and estimated CKD cases attributable to current smoking (A), SBP (B), DBP (C), and BMI (D). CKD cases represented as solid bars and circles represent mean values. The mean (SE) of each risk factor distribution at each time point was standardized by age, sex, and urban or rural distribution using the 2010 Chinese Population Census data as the standard. BMI, body mass index; CKD, chronic kidney disease; DBP, diastolic blood pressure; SBP, systolic blood pressure.

According to the projections of future trends in Figure 2, the means of SBP, DBP, and BMI would continue to increase, while the proportion of current smoking would continue to decrease in the future 20 years. The average SBP in adults was 115 (SE, 0.20) mm Hg in 1991, which increased to 125 (SE, 0.16) mm Hg in 2011, and would increase to 135 (SE, 0.22) mm Hg in 2031. The increase in SBP was estimated to cause 1.7 (95% CI, 1.6–1.8) million CKD events from 1991 to 2011, and would be responsible for another 3.0 (95% CI, 2.8–3.1) million cases in the future 20 years. The prediction of future trends suggested that high SBP would have a PAF of 9.6% (95% CI, 5.0–12.8%) and be responsible for 8.8 (95% CI, 8.3–9.2) million CKD events in 2031. The average DBP in adults was 74 (SE, 0.12) mm Hg in 1991, which increased to 79 (SE, 0.1) mm Hg in 2011, and would increase to 84 (SE, 0.14) mm Hg in 2031. The increase in DBP was estimated to result in 1.5 (95% CI, 1.4–1.6) million CKD events from 1991 to 2011, and would result in another 2.9 (95% CI, 2.8–3.1) million CKD cases from 2011 to 2031, high DBP in 2031 was estimated to have a PAF of 7.4% (95% CI, 2.7–11.6%) and be responsible for 6.8 (95% CI, 6.4–7.1) million CKD events. The mean BMI in adults increased from 21.67 (SE, 0.03) kg/m^2^ in 1991 to 23.92 (SE, 0.04) kg/m^2^ in 2011, the number of CKD cases attributable to high BMI was 2.4 (95% CI, 2.3–2.5) million and 5.4 (95% CI, 5.1–5.6) million in 1991 and 2011, respectively. In 2031, the average BMI would increase to 25.53 (SE, 0.06) kg/m^2^ and would be have a PAF of 9.5% (95% CI, 4.7–13.6%) and accounted for 8.7 (95% CI, 8.2–9.1) million CKD events. The frequency of current smoking in adults decreased from 33.7% in 1991 to 26.5% in 2011, and would continue to decrease to 20.4% in 2031. A decline trend of current smoking was estimated to result in 2.1 (95% CI, 2.0–2.2) million fewer CKD events during 1991–2011, and could prevent 2.0 (95% CI, 1.9–2.1) million CKD cases during 2011–2031, but current smoking would still be responsible for 5.9 (95% CI, 5.6–6.2) million CKD events with an estimated PAF of 6.5% (95% CI, 4.5–8.7%) in 2031.

## DISCUSSION

The current study examined a variety range of lifestyle risk factors among a large and representative adult Chinese population. Multiple modifiable risk factors including current smoking, high TG, high SBP, high DBP, high BMI, high blood glucose, and low HDL-C accounted for a large number of CKD events in China. We observed modest improvement in the smoking, which may have slowed the rapid increase in the burden of CKD. Nevertheless, the smoking rate still fell short of the optimal goal, and this modest improvement could not counteract the increasing burden of CKD due to unfavorable concurrent changes in blood pressure and BMI.

Current smoking is the first leading risk factor of CKD. China signed the World Health Organization Framework Convention on Tobacco Control in 2003 and came into effect in 2005. Despite the rates of smoking having reduced and potentially mitigating the increase in CKD, current smoking rates are still high. According to the Chinese Center for Disease Control and Prevention, the smoking rate among people aged more than 15 years in China was 26.2% in 2018, which is still far from the goal of reducing the smoking rate to 20% in 2030. In 2015, 11.5% of global deaths (6.4 million) were attributable to smoking worldwide, of which 52.2% took place in four countries (China, India, the United States, and Russia). Smoking was ranked among the five leading risk factors by DALYs in 109 countries and territories in 2015, rising from 88 geographies in 1990.^15^ The systematic analysis for the GBD 2017 showed that smoking has been the first and second risk factor for death and DALYs in China, respectively.^4^ Previous prospective cohort studies found that smoking was associated with an increased risk of incident CKD in the adult general population independent of traditional risk factors, such as age, hypertension, and diabetes.^10^^,^^16^^,^^17^ Furthermore, current smokers have a greater risk for CKD than former smokers (RR 1.34 vs 1.15).^10^ However, the awareness of smoking is harmful to health and the intention of smoking cessation among Chinese adult smokers are generally low. The rate of smoking cessation was 16.9% in 2010, 14.4% in 2015, and 20.1% in 2018. Our study estimated the decline trend of current smoking could prevent 2 million CKD cases during 2011–2031, but current smoking would still be responsible for 5.9 million CKD events in 2031. To further lightening the burden of CKD, smoking control should be further strengthened.

The increasing upward trends in SBP and DBP was consistent with Chinese national surveillance data showing a continuous increase in the prevalence of hypertension over the past decades: The prevalence of high blood pressure was 5.1% in 1959, 7.7% in 1979, 13.6% in 1991, 17.7% in 2002, 29.6% in 2010, and 37.2% in 2017.^18^^–^^21^ According to the 2017 American College of Cardiology/American Heart Association clinical practice guideline, the prevalence of hypertension among Chinese adults was 46.4%, similar to that in United States.^22^^,^^23^ In 2017, high blood pressure ranks as the top and second risk factor for CKD burden in east Asia and worldwide, respectively.^2^ Furthermore, hypertension accounted for the largest proportion of death in China.^4^ The increase in the prevalence of hypertension may be partly due to the dyslipidemia and higher BMI. Population-based epidemiological studies have strongly indicated a causal relationship between dyslipidemia or BMI and risk of incident hypertension.^24^^–^^27^ The estimated PAF of 6.3% and 4.2% of high SBP and DBP implied that about 2 to 3 in 50 CKD events in China might be prevented if SBP and DBP could be managed to the theoretical minimum level of 115 and 75 mm Hg.

During the last decades, the rapid transition to a Western dietary pattern has led to rapid increase in the prevalence of BMI, dyslipidemia, and high plasma glucose in China. In 2014, the number of obese people in Chinese men and women were 43.2 million and 46.4 million, respectively, ranking first in the world. The worldwide ranking of the number of severely obese individuals has moved China from 60th place for men and 41st place for women in 1975 to second for both men and women in 2014, second only to America.^28^ The high BMI was the third risk factor of CKD quantified in GBD and accounted for 9.5% of the age-standardized rate of CKD DALYs in 2017.^2^ While the data on the etiological effect of BMI used in this study was derived from Japan and Korea populations, these populations are Asian and similar to the Chinese population.^29^ Hence, our estimated CKD burden attributable to high BMI in China should be relatively accurate. In additional, the high TG and low HDL-C were the two major types of dyslipidemia in Chinese adults. The prevalence of high TG and low HDL-C was 11.9% and 7.4% in 2002, which increased to 12.17% and 15.31%, respectively, in 2010.^30^ Our study found that high TG was the second risk factor of CKD burden in China. Type 2 diabetes is a growing epidemic in China and was rare in China in the 1980s, with an estimated prevalence of 0.67%.^31^ In subsequent national surveys conducted in 1994, 2000 to 2001, 2007 to 2008, and 2010 to 2011, the prevalence of diabetes was 2.5%, 5.5%, 9.7%, and 11.6%, respectively.^32^^–^^34^ Starting from 2011, the percentage with CKD related to diabetes exceeded the percentage with CKD related to glomerulonephritis in China, and the gap between them increased progressively. In 2015, the percentage of the Chinese population with CKD related to diabetes and to glomerulonephritis was 1.23% and 0.91%, respectively.^35^ The above data imply that CKD events associated with high BMI, high TG, high plasma glucose, and low HDL-C will continue to increase in the future.

CHNS is the only large-scale longitudinal study of its kind in China and has a high response rate (88%) and originality.^11^ The randomly selected households in nine provinces have provided a broad-based indication of the trends taking place in China. To our knowledge, the current study is the first population-level long-term analysis of the CKD burden associated with lifestyle factors in China using comparable methods. Our estimates were based on the most recent and best available evidence on risk factor-CKD associations, derived primarily from meta-analyses.

However, there are still some limitations in our study. Unhealthy diet is an important risk factor for non-communicable diseases and death. In 2017, 11 million deaths and 255 million DALYs around the globe were attributable to dietary risk factors.^36^ Although some previous studies have shown some dietary factors, including the increase in sodium, animal protein, red meat, and sugar sweetened beverages intake and the decrease in fruit, vegetables, and fiber intake, were associated with higher risk incidence CKD, the relevant studies based on health population are rare and the results remain controversial.^7^^,^^37^^–^^40^ Therefore, there was no evidence-based RR on dietary factors contribute to CKD, so we couldn’t evaluate the trends of dietary factors in our study. Second, CKD is a chronic complex disease and likely caused by multiple factors. CKD events attributable to individual risk factors often overlap, and the total CKD events attributable to all risk factors cannot simply be summed. Third, the etiological effects of risk factors on CKD that were used in this study were derived from meta-analyses, which primarily contain studies from non-Chinese populations. Future estimations should base on meta-analyses of Chinese populations when more data become available. Finally, we did not consider aging or population growth in our time-trend analysis of CKD burden. We applied CKD events in 2011 to all PAFs in different waves to estimate the time trend of attributable CKD burden. This may have overestimated the CKD burden before 2001 but underestimated future CKD burden, because the Chinese population is aging. Our projection of CKD burden associated with individual risk factors might be an underestimation, as our standardization and estimates were on the basis of the population proportion and number of CKD events in 2011.

In conclusion, although decreased smoking prevalence contributed to some reduction in CKD burden, the current levels of smoking remain suboptimal, and smoking is the leading factor contributing to the CKD burden in China. High TG, high blood pressure, high BMI, high plasma glucose, and low HDL-C were also important contributors. It is still necessary to take corresponding measures to quit smoking and control blood pressure, plasma glucose, blood lipids, and weight. Our findings can be used to help inform policy strategies for CKD prevention at the population level.

## SUPPLEMENTARY MATERIAL

eTable 1. Distribution of Chinese populations according gender, residence, and age in 2010
