# Supplementary material for: Risk Assessment for Longitudinal Trajectories of Modifiable Lifestyle Factors on Chronic Kidney Disease Burden in China: A Population-based Study
Source: J Epidemiol. 2022 Oct 5;32(10):449–55. doi: 10.2188/jea.JE20200497 (PMC9424184; doi:10.2188/jea.JE20200497)
Supplement: Supplementary file 1 [file je-32-449-s001.pdf]

eTable 1. Distribution of Chinese populations according gender, residence, and age in 2010

Sex	Residence	Age, years	Number of populations
Male	Urban	18–39	8,9212,538
		40–59	60,074,645
		60–69	12,657,180
		≥70	9,721,184
	Rural	18–39	107,773,253
		40–59	99,369,085
		60–69	28,649,258
		≥70	20,207,962
Female	Urban	18–39	85,875,852
		40–59	56,815,669
		60–69	13,059,648
		≥70	10,875,661
	Rural	18–39	104,919,180
		40–59	97,005,117
		60–69	27,184,060
		≥70	23,262,017
		total	846,662,309
